# Strengthening epidemiologic investigation of infectious diseases in Korea: lessons from the Middle East Respiratory Syndrome outbreak

**DOI:** 10.4178/epih/e2015040

**Published:** 2015-09-16

**Authors:** Changhwan Lee, Moran Ki

**Affiliations:** 1 Korea Centers for Disease Control and Prevention, Cheongju, Korea; 2 Department of Cancer Control and Policy, Graduate School of Cancer Science and Policy, National Cancer Center, Goyang, Korea

**Keywords:** Middle East Respiratory Syndrome, Coronavirus infections, Epidemiologic investigation, Infectious diseases, Korea, Outbreak

## Abstract

The recent outbreak of Middle East Respiratory Syndrome (MERS) coronavirus infection in Korea resulted in large socioeconomic losses. This provoked the Korean government and the general public to recognize the importance of having a well-established system against infectious diseases. Although epidemiologic investigation is one of the most important aspects of prevention, it has been pointed out that much needs to be improved in Korea. We review here the current status of the Korean epidemiologic service and suggest possible supplementation measures. We examine the current national preventive infrastructure, including human resources such as Epidemic Intelligence Service officers, its governmental management, and related policies. In addition, we describe the practical application of these resources to the recent MERS outbreak and the progress in preventive measures. The spread of MERS demonstrated that the general readiness for emerging infectious diseases in Korea is considerably low. We believe that it is essential to increase society’s investment in disease prevention. Fostering public health personnel, legislating management policies, and establishing research centers for emerging infectious diseases are potential solutions. Evaluating international preventive systems, developing cooperative measures, and initiating improvements are necessary. We evaluated the Korean epidemiologic investigation system and the public preventive measures against infectious diseases in light of the recent MERS outbreak. We suggest that governmental authorities in Korea enforce preventive policies, foster the development of highly qualified personnel, and increase investment in the public health domain of infectious disease prevention.

## INTRODUCTION

Korea recently experienced an unprecedented outbreak of Middle East Respiratory Syndrome (MERS), in which it became the country with the second most confirmed patients worldwide, after Saudi Arabia. During this outbreak, which caused massive economic damage and social disruption throughout the country, the importance of preventive measures against the spread of infectious diseases from abroad, such as quarantine and epidemiologic investigations, became increasingly clear. The purpose of conducting epidemiologic investigations when an outbreak of an infectious disease such as MERS occurs is to promptly confirm the outbreak and to identify the causes and sources of infection. Ultimately, the goal is to prevent the spread of the infectious disease. The importance of epidemiologic investigations lies not only in the fact that they effectively prevent the spread of the ongoing infectious disease, but also in the fact that they provide information allowing the prediction and prevention of potential outbreaks in the future. The effective implementation of epidemiologic investigations requires the presence of a solid infrastructure including a national response system for infectious diseases, sufficient investment in public health institutions, high-quality operational management, and, above all, policies that support the training and retention of highly qualified personnel.

Modern epidemiologic investigations of infectious diseases have more than 150 years of history, dating from the epidemiologic investigation of cholera that was performed by John Snow (1813-1853), known as the father of epidemiology. Epidemiologic investigations employ a rational and scientific methodology that integrates logical reasoning to identify the cause of an outbreak. Although epidemiologic investigations can be conducted in different ways depending on the situation, the principles are the same. The first step is verifying that an outbreak has taken place and measuring its size, the second step involves conducting descriptive epidemiological analyses of the epidemic, the third step is formulating hypotheses based on those analyses, the fourth step involves examining those hypotheses using analytical epidemiologic methodologies, and the fifth step comprises evaluation and communication [1].

Korea suffered a large-scale MERS outbreak in 2015, and critics have suggested that the epidemiologic investigation was not properly carried out. In this study, we provide a chronological review of the epidemiologic investigation of the MERS outbreak as well as suggestions about how to strengthen epidemiologic investigations in the future.

## CRISIS LEVELS IN THE MANAGEMENT OF INFECTIOUS DISEASES

The official Korean manual for the management of infectious diseases establishes four crisis levels: attention, caution, alert, and severe. ‘Attention’ refers to situations where no patients of a given disease have been confirmed in Korea, but it is possible for the disease to be imported into Korea, since some patients have been identified in other countries. This level involves preparing for an outbreak in the case of an influx of patients to Korea by establishing preparedness and response plans, specifying the roles of the relevant institutions, and providing education programs for the personnel who would be involved. In 2014, the Public Health Emergency Response Group held a symposium on MERS, and the Korea Centers for Disease Control and Prevention (KCDC) prepared a testing system, published the 2014 MERS management guidelines, and trained public health personnel to handle MERS cases. The 2014 management guidelines stated that “these guidelines can be applied in situations where the national infectious disease crisis level is ‘attention’ or ‘caution’, and can be continuously revised as the situation changes” [2].

In the case of the recent MERS outbreak, the most important element of the ‘attention’ crisis level was the early detection of MERS cases with the goal of treating them in isolation. However, the early clinical identification of MERS cases is difficult because the symptoms of MERS are non-specific [3]. Therefore, the most important factor in the early diagnosis of MERS is implementing a surveillance program to check the travel histories of patients with respiratory symptoms. When suspected MERS cases are identified, they should be promptly isolated, the medical staff should wear protective equipment, and clinical specimens should be examined in the early stage of the illness. The guidelines specify that when the first confirmed case is identified, the KCDC should elevate the infectious disease crisis level from ‘attention’ to ‘caution’. When the crisis level is raised to ‘caution’, a task force reporting to the director of the KCDC should be established and put in charge of managing the response to MERS. These actions were taken on May 20, 2015, when the first confirmed MERS case was identified [4].

## THE PRINCIPLES OF EARLY EPIDEMIOLOGIC INVESTIGATION

When a confirmed case is identified, an epidemiologic investigation is carried out by the Epidemic Intelligence Service (EIS) officers of the KCDC. At this time, a very careful and thorough epidemiologic investigation should be carried out and proper preventive measures should be put in place. When dealing with emerging infectious diseases whose characteristics are not yet well understood, it is safest to implement maximal precautionary measures.

The first step of an epidemiologic investigation is verifying that an outbreak has taken place and measuring its size of the outbreak, following these steps:

(1) Suspected or confirmed cases should be correctly identified. All suspected cases should be identified, even though they have not yet been confirmed.
(2) Suspected cases should be examined to ascertain whether they are suffering from a single disease, even if their symptoms are only roughly similar. In the initial stages of an epidemiologic investigation, the case definitions are usually determined by reviewing the presentation of the initial cases due to the absence of established guidelines. A rough case definition is initially used because it is difficult to apply precise case definitions in the early stages of an outbreak, and case definitions can be made clearer once more information is gathered. Additionally, the quantity of cases should be estimated, and cases should be classified as laboratory-confirmed cases and probable cases who have not been confirmed by laboratory testing, but are relevant from an epidemiological standpoint [2,5].
(3) After determining the extent of the incidence of new cases, a decision must be made about whether an outbreak has occurred. This decision must be made based on the expected number of cases, as indicated by previous information. Comparison with previous information is especially necessary when a change in incidence has been noted over a relatively long period of time. However, in some cases, the presence of even one patient with a given disease should be considered as an indication that an imminent outbreak is highly likely. This is the case for emerging infectious diseases that are completely new to Korea, as well as for existing infectious diseases, such as the plague, which have not been present in Korea for a long period of time but have the potential to result in an outbreak.

After verifying the presence of an outbreak and estimating its size in the first stage of the epidemiologic investigation, it is necessary to determine what would constitute an appropriate response. It is also important to collect, analyze, and store all possible specimens in order to identify the cause of the outbreak.

## ACTIONS TO BE TAKEN WHEN AN OUTBREAK IS IDENTIFIED

If it is determined that an outbreak has taken place, preventive measures should be implemented. Depending on the type of the infectious disease and the timing of the outbreak, the prioritization and the specific details of the preventive measures will vary. In instances of single exposures, as in cases of food poisoning, the mode of transmission should be identified by preserving suspected foods or ensuring that the site of transmission remains intact. However, if the disease spreads through person-to-person transmission, prompt and active preventive measures need to be taken. For infectious diseases spread by person-to-person transmission, each patient is a source of infection, and it is therefore important to identify suspected cases as soon as possible and to treat them in isolation. Some diseases are transmitted during the incubation period, while other diseases are transmitted only after the onset of symptoms. The period of infectiousness as well as the natural history of a disease should be taken into consideration when implementing preventive measures. Infectious diseases such as the common cold and influenza are transmitted before the onset of symptoms, making it difficult and inefficient to identify and quarantine people who have been exposed. However, severe acute respiratory syndrome, and MERS are known to be infectious only after the onset of symptoms, not during the incubation period; therefore, actively identifying contacts to be monitored and close contacts to be quarantined can be an effective measure for preventing the spread of this disease [6,7]. In order to carry out such measures, it is necessary to obtain information about the contact history of confirmed cases. It is also important to properly disinfect areas where confirmed cases have been present in order to eliminate the possibility of further infection [6].

This article focuses on the process of epidemiologic investigations, and therefore does not describe preventive measures. The following items must be carried out during an epidemiologic investigation: 1) determining the period of infectiousness (for MERS, after the onset of symptoms); 2) tracing the movement of the cases during the period of infectiousness; 3) identifying contacts of the cases before quarantine; 4) if the confirmed cases visited any hospitals, blocking the spread of the infection in those hospitals by urging them to implement prompt preventive measures; 5) after identifying the major paths of cases, directly interviewing the cases if possible to verify their movements, supplementing their list of contacts, and, if necessary, obtaining consent for checking credit card details; 6) further confirming that the records of their movement are complete by checking credit card details; 7) checking whether contacts of the case show the symptoms of the disease and, if so, performing the same investigation that was performed for the first case; 8) when necessary, identifying any missing contacts by checking surveillance cameras; 9) when necessary, collecting environmental specimens and performing laboratory examinations; and 10) when necessary, inspecting hospital facilities to ensure that preventive measures are taken against the outbreak.

## BRIEF DESCRIPTION OF THE MERS EPIDEMIOLOGIC INVESTIGATION, KOREA, 2015

In this section, we describe the actual epidemiologic investigation performed during the 2015 MERS outbreak in Korea, focusing on key events in chronological order. Cases that did not fit into our knowledge and expectations appeared over the course of the outbreak, and the investigative methods evolved accordingly. Additionally, the definitions for cases and contacts, as well as management policies, were changed over the course of the outbreak.

### May 20-May 25, 2015: investigation carried out according to the 2014 MERS management guidelines

On May 20, the first MERS case (Patient zero) was confirmed, and the KCDC followed the 2014 MERS management guidelines [2] regarding the definition of close contacts and the assignment of investigative roles to hospitals. A close contact was defined as a person who had physical contact with either a confirmed case or a suspected case, or a person who stayed within a two-meter radius of a case for more than one hour in the same space. Sixteen medical staff members were quarantined and actively monitored based on this definition. Although suspected cases were defined as anyone who had clinical, radiological, histological, or pathological pulmonary parenchymal disease, such as pneumonia or acute respiratory distress syndrome, in practice, the presence of a fever over 38°C was used to identify suspected cases. Closed-circuit television (CCTV) records were analyzed to objectively determine the level of exposure in the affected hospitals during the epidemiologic field investigation. CCTV analysis is commonly employed in forensic science, but this was the first time that this method was applied to the epidemiologic investigation of an infectious disease in Korea.

### May 25-June 3: investigation carried out according to the third edition of the MERS management guidelines

On May 23rd, the fourth case (the daughter of the third confirmed case) asked the health authorities for a MERS confirmatory test and quarantine after observing that her body temperature was 37.9°C, but the health authorities did not accept this request. This case was confirmed on May 26. Also, on the same day, a physician who treated the Patient zero (#1) for less than five minutes was confirmed to have MERS. Thus, the criterion for the temperature of a suspected case was lowered to 37.5°C, and myalgia, chills, and diarrhea were also included as suspicious symptoms in the new guidelines. Additionally, the one-hour criterion for the duration of contact was no longer considered sufficient for identifying close contacts, so this criterion was eliminated in the new guidelines. Although it normally takes some time to establish and distribute new guidelines, these new criteria were immediately established by the epidemiologic investigators and applied in the field.

### June 3-June 7: investigation carried out according to edition 3.2 of the MERS management guidelines

On May 28, the sixth confirmed MERS case was identified. This case had not been previously classified as a close contact. The health authorities implemented an overall reinvestigation of Pyeongtaek St. Mary’s Hospital in order to find evidence of close contact with the Patient zero. Furthermore, on May 31, the joint public-private Expert Committee on the MERS-Coronavirus Outbreak Investigation was established. The reinvestigation included analyzing CCTV footage in the hospitals, determining the spatial distribution of all cases who were eventually diagnosed with MERS using hospital maps, obtaining a list of all medical staff, hospitalized patients, and caregivers reevaluating exposure and reclassifying close contacts, investigating the hospital ventilation system, and collecting and examining environmental specimens. Despite this comprehensive reinvestigation, it was difficult to adequately identify close contacts, and MERS cases continuously occurred. It became clear that considering only droplets as a mode of transmission, as was initially thought, was not enough to account for the observed patterns of MERS occurrence. Consequently, the diffusion of air currents within the wards of the Pyeongtaek St.Mary’s Hospital was studied and an in-depth investigation of medical staff members was carried out in order to identify the mode of transmission of the disease. These considerations were also reflected in the evolving MERS management guidelines; thus, the definition of suspected cases was expanded to include employees, patients, caregivers or visitors with symptoms such as fever or shortness of breath who were in a hospital where cases of MERS occurred within 14 days of the onset of symptoms of the suspected case. Also, after the public was informed on June 5 that Pyeongtaek St. Mary’s Hospital was the locus of the outbreak, personnel from the Health Insurance Review and Assessment Service started to support the epidemiologic investigation. Their role was to help check the hospital visit histories of cases who had been hospitalized anywhere in Korea and to obtain hospital visit histories as required for the epidemiologic investigation. This step was taken because relying only on case statements during the epidemiologic investigation resulted in missing hospitals that cases had actually visited. Therefore, in order to ensure that no hospitals were missed, Drug Utilization Review information was utilized to obtain hospital and pharmacy visit histories for use in the epidemiological investigation. This was also a method that was not used in the initial epidemiologic investigation.

### June 7-present: investigation carried out according to edition 3.3 of the MERS management guidelines

On June 7, all information about the hospitals where MERS cases had occurred or MERS cases had visited was disclosed, and people who had visited those hospitals during the period when MERS cases were present were contacted and informed that they should notify the call center if they had any symptoms of MERS. Edition 3.3 of the MERS management guidelines was also published [8]. The contents of edition 3.2 of the MERS management guidelines were preserved regarding the definitions of cases and close contacts. Since the outbreak had already lasted for some time, the revised edition included considerable information about secondary issues, such as treatment protocols, criteria for determining when confirmed cases have completely recovered, protocols for lifting the quarantine of those who had been isolated in their homes, and procedures for dealing with the death of MERS cases. At this time, it was possible to implement a cohort isolation in Daechung Hospital and Konyang University Hospital, based on lessons from the outbreak in Pyeongtaek St. Mary’s Hospital.

In the early phase of the outbreak, the concept of cohort isolation was very unfamiliar and the ethical and legal grounds for cohort isolation were not robust. Moreover, general compliance was low. However, as the outbreak caused economic losses and social disruption, the general public reached a consensus that made it easier to implement drastic measures to prevent the further transmission of MERS. The support of local governments was also very helpful. Local governmental institutions agreed that timely and proper preventive measures were the best policy for handling the outbreak. In particular, the events that occurred on Jeju Island are worthy of close attention. A probable MERS case traveled to Jeju Island while symptomatic. Discrepancies were observed between his statements and those of his companions. Additional measures were taken due to these inconsistencies, including location tracking using the base stations of mobile phones and checking credit card bills. In addition, the automative navigation system of a rental car that a MERS case used while traveling was used to track that case’s movements. These were new methods that had not been used in previous epidemiological investigations.

## CHALLENGES IN THE EPIDEMIOLOGICAL INVESTIGATION

### The role of Epidemic Intelligence Service officers

EIS officers check the contacts of a confirmed case during an investigation and establish the standards used to designate persons subject to epidemic prevention measures based on the level of contact. It is also the role of the health department and public health centers in a given city or province to take preventative measures against epidemics, such as locating persons subject to preventive measures, calling them individually, checking if they have any symptoms, performing tests if necessary, and implementing tracking and management procedures such as quarantine and active monitoring. However, many situations exist in which these roles are not clearly distinguishable. If an outbreak occurs in a hospital, as occurred in this MERS outbreak, it is very difficult for public health centers to implement quarantines or to track and manage contacts who are hospitalzed patients without the cooperation of hospitals and clinics, even though EIS officers officially determine the criteria used to designate persons subject to preventative measures based on the extent of their contact with the infected case.

In addition, it is extremely difficult for EIS officers to make decisions alone in this context, since EIS officers have no legal power to order the temporary suspension of services in a hospital where cases occurred, even when it is necessary to take urgent measures to prevent an epidemic. Moreover, the suspension of services in a large hospital can cause serious violations of the rights of other patients, who have no connection with the epidemic, to be treated. These issues were continuously raised during the MERS outbreak. Moreover, difficulties were encountered in determining which investigator had to make a decision due to the absence of a decision-making system enabling the real-time discussion of several issues, which caused this process to be delayed or omitted.

### The protection of personal information

In order to identify close contacts of the MERS cases and to take measures to prevent an epidemic, it is necessary for the investigation to access the personal data and medical records of cases and their contacts. During the MERS outbreak, the epidemiological investigation encountered many difficulties due to the absence of regulations enabling the investigators to carry out the investigation without coming into conflict with the Personal Information Protection Act. Additionally, many cases continued their daily lives as usual while symptomatic until they were confirmed to be infected; this interval ranged from as short as one to two days to as long as over one week. In such a situation, relying only on interviews with the case to identify their contacts is likely to miss crucial information. Cases cannot remember everything, and some cases were in a critical condition that rendered interviews impossible. Moreover, cases sometimes do not disclose their contacts for personal reasons. During the MERS outbreak, contacts were missed for these reasons, and some of those who were missed eventually became infected, resulting in the risk of a large epidemic. Therefore, cases’ use of medical institutions was checked using data from the Health Insurance Review and Assessment Service, and cases’ movements were established by cross-referencing their statements with CCTV records and details regarding their use of mobile phones and credit cards. Checking CCTV records currently requires assistance from technical specialists, as well as consent from the person concerned. Tracking the location of mobile phones and obtaining information about their credit card use require the agreement of the case, guardian of the case, and/or the person living with the case. A set of measures should be established to ensure that the goals of an epidemiological investigation are accomplished rapidly while protecting civil rights and personal information.

### Protection of epidemiological investigators, including Epidemic Intelligence Service officers

As observed in this MERS outbreak, when a case occurs in a hospital, the hospitals and clinics must be considered to be contaminated. Moreover, interviewing the case constitutes close contact. Therefore, epidemiological investigators should be fully prepared to take infection prevention measures before they participate in the investigation. However, in the beginning of the epidemiological investigation of MERS, level D protective gear was not provided to epidemiological investigators, and the epidemiological investigation team was set up inside the affected hospital. If any of the investigation personnel are infected, all members of the team should be quarantined. This would lead to a major crisis and a vacuum in the epidemiological investigation. It is therefore extremely important to ensure that the investigation personnel do not become infected.

### Inadequacies in the system of compensation for quarantine measures

When the epidemiological investigation confirms that a person has been in close contact with an infected case, that person is subject to quarantine measures, such as home quarantine or active monitoring. Facilities including hospitals and clinics are subject to measures such as temporary shutdown according to the quarantine guidelines. However, no guidelines existed regarding adequate compensation for the losses incurred by affected persons and facilities following quarantine measures. This led to noncooperation from the persons and facilities in question, damaging the effectiveness of the quarantine measures.

### Close cooperation among public health departments in central and local government and public health centers

In this outbreak of MERS, the disease spread to several regions in a short time, making close cooperation between the affected municipalities and the central government very important. However, disclosure of information from the central government was delayed and confirmatory test methods were not quickly transferred to the Research Institute of Public Health and Environment of each municipality, resulting in a late and discordant response from the municipalities.

## DISCUSSION

### Measures to strengthen epidemiological investigations

Several problems mentioned above should be solved in order to ensure that future epidemiological investigations will be implemented successfully. This section contains some suggestions for improving subsequent epidemiological investigations in Korea.

### Review and amendment of related laws

According to Article 2, No. 17 of the Infectious Disease Control and Prevention Act, the term “epidemiological investigation” refers to the activity of investigating cases involving patients infected by an infectious disease, patients suspected of an infectious disease, and carriers of the pathogen, as well as tracing the sources of their infection, in order to quarantine such infectious diseases and to prevent their spread, and the investigation of adverse events to vaccinations, if any such cases occur.

In Article 12, No. 1 of the Enforcement Decree of the Infectious Disease Control and Prevention Act, the contents of an epidemiological investigation are clearly stipulated to involve the followings: (1) the personal information of patients infected by an infectious disease; (2) the date and place of their infection; (3) the origin and route of infection; (4) the medical records of the patients infected by the infectious disease; (5) other factors related to examining the causes of the infectious disease.

In the Article 14 of the Enforcement Decree of the Infectious Disease Control and Prevention Act, the method of an epidemiological investigation is stipulated to involve the following steps: (1) questionnaires and interviews; (2) collecting and testing clinical specimens; (3) collecting and testing specimens from the environment; (4) collecting and testing specimens from vectors of infectious diseases, such as insects and animals; (5) investigating medical records and interviewing doctors.

However, these laws and decrees do not contain any content regarding extremely urgent situations in which the epidemiological investigation is directly linked to quarantine measures, as occurred in this MERS outbreak. For example, no phrase approves measures for checking personal information, including securing and analyzing CCTV records, the location tracking of mobile phones, and checking the details of credit card use. These measures are necessary for establishing the movements of an infected case in order to identify a case’s contacts, although the Personal Information Protection Act conflicts with quarantine measures that may be required. Clear stipulations approving a flexible response are necessary, since future epidemiological investigations may vary in terms of content, scope, and method, depending on the characteristics of future outbreaks.

### Reinforcing professional personnel in epidemiological investigations and strengthening their role

Considerable criticism was raised regarding inadequacies in the current system of epidemiological investigations during this MERS outbreak. Accordingly, the National Assembly of Korea amended the system of epidemiological investigations and the Infectious Disease Control and Prevention Act. The amended law was immediately enforced when it was promulgated on July 6, 2015, and the revised system of epidemiological investigations is scheduled to be put in place in January 2016. Regarding the reinforcement of the personnel of epidemiological investigations, Article 60, No. 2 of the Infectious Disease Control and Prevention Act stipulates that “More than 30 epidemiological investigation officers shall be assigned to the Ministry of Health and Welfare and more than two epidemiological investigation officers shall be assigned to each city and do (province) to carry out responsibilities concerning the epidemiological investigation of infectious diseases. Provided, a mayor/do administrator may appoint an epidemiological investigation officer in a si/gun/gu (city, county, or district) when necessary to carry out responsibilities concerning the epidemiological investigation”. Regarding the authority of epidemiological investigation officers, it stipulates that “epidemiological investigation officers may temporarily take measures to implement each item in Article 47, No. 1 in an urgent situation where the infectious disease is expected to become epidemic, seriously damaging public health unless immediate measures are taken.” The measures in Article 47, No. 1 include “to carry out the following measures in places where infected patients present or places that have been deemed to be contaminated by the infectious pathogen: a) temporary shutdown, b) making a given place off-limits to the general public, c) restriction of movement inside a given place, and d) other measures necessary to block passage” [9].

It is appropriate that this law was amended, even though the amendments were late. However, it is necessary to establish a new organization for training capable epidemiological investigators in order for this law to have its intended effect. Additionally, a system should be prepared for systematizing and utilizing the experiences of the investigators after training. In addition, epidemiological investigators should be provided with better working conditions, in order to encourage competent specialists to become epidemiological investigators. A specific framework should be established on the basis of discussions about this issue with specialists from different fields.

### Necessity of a research center for emerging infectious diseases

This MERS outbreak made it known to the world that Korea is not ready to respond to emerging infectious diseases adequately. In contrast to Korea’s fame as an information technology powerhouse in the digital age, epidemiological investigations were still being performed in an outdated way, in which official documents had to be sent to obtain cooperation, the results of the investigation were written on paper, typed, and summarized using a word processing program, which need a considerable period of time. This system is especially inefficient in urgent cases, where epidemiological investigators immediately determine the required quarantine measures in order to control the spread of the outbreak quickly. In contrast, this system experienced no problems in cases where epidemiological investigations began at the end of an outbreak such as foodborne outbreak. Moreover, excessive anxiety and fear were spread due to incorrect communication about risks at a time when the results of the epidemiological investigation should have been disclosed not only to the general public, but also to specialists. It brought an unnecessary school shutdown across the country. In order to solve these problems, a permanent institution in which specialists from various fields can prepare for epidemics of infectious diseases should be established through the establishment of a research center for emerging infectious diseases. This institute should closely monitor the current status of diseases worldwide and prepare plans for a response within Korea. In addition, a digital system for epidemiological investigations utilizing various sources of personal information, such CCTV records, the use of navigation systems, credit card use, and prior medical records (including electronic medical records) needs to be prepared, as well as a system in which the contents of epidemiological investigations can be shared in real time between specialists and policymakers. It is also imperative for specialists in risk communication to communicate with other specialists, citizens, and educators in each stage of all epidemiological investigations.

## CONCLUSION

The response to the recent MERS outbreak in Korea demonstrated inadequate preparedness for emerging infectious diseases. Subsequent MERS outbreaks could reoccur at any point unless the problems raised by this outbreak are solved. Bill Gates has warned that the greatest threat to mankind in the 21st century is emerging infectious diseases, not nuclear weapons [10]. Korea should be well prepared to respond to emerging infectious diseases, utilizing this crisis as a chance for future improvement before it is too late.
