# Supplementary material for: Strengthening epidemiologic investigation of infectious diseases in Korea: lessons from the Middle East Respiratory Syndrome outbreak
Source: Epidemiol Health. 2015 Sep 16;37:e2015040. doi: 10.4178/epih/e2015040 (PMC4616012; doi:10.4178/epih/e2015040)
Supplement: Supplementary file 1 [file epih-37-e2015040-supplementary.pdf]

한국의 감염병 역학조사 강화방안: 중동호흡기증후군 유행 경험에서의 교훈

이창환 · 기모란

질병관리본부 역학조사과, 국립암센터 국제암대학원대학교 암관리정책학과

Strengthening epidemiologic investigation of infectious diseases in Korea: lessons from the Middle East respiratory syndrome outbreak

Changhwan Lee, MD · Moran Ki, MD, PhD

Department of epidemic intelligence service, Korea Centers for Disease Control and Prevention, Cheongju, Korea

Department of Cancer Control and Policy, Graduate School of Cancer Science and Policy, National Cancer Center, Goyang, Korea

Corresponding author: Moran Ki

E-mail: moranki@naver.com

ORCID

Changhwan Lee, http://orcid.org/ 0000-0001-9574-4333

Moran Ki, http://orcid.org/ 0000-0002-8892-7104

Running title: Strengthening epidemiologic investigation of infectious diseases

Abstract

The recent outbreak of Middle East respiratory syndrome (MERS) coronavirus infection in Korea resulted in large socioeconomic losses. This provoked the Korean government and the general public to recognize the importance of having a well-established system against infectious diseases. Although epidemiologic investigation is one of the most important aspects of prevention, it has been pointed out that much needs to be improved in Korea. We review here the current status of the Korean epidemiologic service and suggest possible supplementation measures.

We examine the current national preventive infrastructure, including human resources such as Epidemic Intelligence Service officers, its governmental management, and related policies. In addition, we describe the practical application of these resources to the recent MERS outbreak and the progress in preventive measures.

The spread of MERS demonstrated that the general readiness for emerging infectious diseases in Korea is considerably low. We believe that it is essential to increase society's investment in disease prevention. Fostering public health personnel, legislating management policies, and establishing research centers for emerging infectious diseases are potential solutions.

Evaluating international preventive systems, developing cooperative measures, and initiating improvements are necessary.

We evaluated the Korean epidemiologic investigation system and the public preventive measures against infectious diseases in light of the recent MERS outbreak. We suggest that governmental authorities in Korea enforce preventive policies, foster the development of highly qualified personnel, and increase investment in the public health domain of infectious disease prevention.

Key words; MERS, Epidemiologic investigation, Infectious Diseases, Korea, Outbreak

찾아보기말: 중동호흡기증후군, 역학조사, 감염병, 한국, 유행

서론

최근 우리나라는 전례 없는 중동호흡기증후군(Middle East respiratory syndrome, MERS; 메르스) 유행을 겪었고, 사우디아라비아 다음으로 전 세계적으로 메르스 확진자가 많은 나라가 되었다. 이번 유행을 겪으면서, 전 국가적으로 막대한 사회경제적 피해가 발생함에 따라 해외 유입 감염병에 대한 검역과 그 확산 방지를 위한 방역 및 역학조사의 중요성이 대두되었다. 메르스를 비롯한 감염병이 발생하였을 때 역학조사를 시행하는 목적은 감염병의 유행 여부를 신속하게 판단함과 동시에 유행의 원인 및 감염원을 밝히고, 그 감염 전파 과정을 파악하여 궁극적으로 감염병의 확산을 방지하는 것이다. 역학조사가 갖는 중요성은 현재 진행 중인 감염병의 유행을 효과적으로 방역하는 것뿐만 아니라, 추후에 발생 가능한 질병의 유행을 사전에 예측하고, 예방할 수 있는 능력을 갖추는데 필수적인 정보들을 제공해주는 것이다. 이러한 역학조사가 효과적으로 이루어지기 위해서는 먼저 국가의 감염병 대응체계를 잘 구축하여 그 기반을 닦고, 이에 대한 충분한 투자와 운영 등의 관리가 뒤따라야 하며, 무엇보다도 질적으로 수준 높은 인적 자원의 양성과 유치에 대한 정책적 뒷받침이 있어야 한다.

감염병 역학조사는 역학의 아버지라 불리는 영국의 존 스노가 첫 콜레라 역학조사를 한 이래로 150년 이상의 역사를 지녔으며, 유행의 원인을 밝히기 위한 논리적인 도구들이 집대성 되어 있는 합리적이며 과학적인 방법론이다[1]. 역학조사는 상황에 따라 다르게 진행 될 수 있지만 원칙적인 부분은 같다. 첫 번째 단계는 유행의 확인과 크기 측정이며, 두 번째 단계는 유행의 기술역학적 분석, 그리고 세 번째 단계는 이를 바탕으로 한 가설의 설정, 네 번째는 분석 역학적 방법론을 이용한 가설의 검증, 그리고 다섯 번째는 평가와 커뮤니케이션이다.

2015년 우리나라에서는 메르스 대유행을 겪으면서 역학조사가 그 역할을 제대로 수행하지 못하였다는 비판이 일었다. 이에 본 글에서는 이번 메르스 유행에서 시행된 역학조사를 시간 순서대로 검토해 보고, 이때 도출된 역학조사의 문제점과 함께 이를 강화할 수 있는 방안을 제시하고자 한다.

감염병 관리 위기 단계

감염병 관리 매뉴얼에 따른 우리나라 감염병 관리 위기 단계는 관심-주의-경계-심각 등의 4단계로 나뉘어진다. 관심단계는 한국에서는 확진환자가 없으나 외국에서 환자 발생이 있어 한국에 유입될 가능성이 있을 때이다. 이때에는 감염병 위기 대비 단계로서 한국에 환자가 유입될 것을 대비하여 대비/대응 지침을 개발하고, 관계 기관별 역할을 명시하고, 관련자들을 대상으로 교육을 시행한다. 한국에서도 2014년도에 '공중보건위기대응 사업단'에서 심포지움을 개최하였으며, 질병관리본부에서는 검사시스템을 갖추고 "2014 중동호흡기증후군 관리 지침"을 발간하고 관계 보건기관 담당자를 대상으로 교육을 시행하였다. 이때 발간한 관리지침에는 "이 지침은 국가 감염병 위기단계 관심 및 주의 단계에서 적용가능하며 상황변화에 따라 지속적으로 개정 가능"하다고 명시되어있다[2].

관심 단계에서 가장 중요한 것은 메르스 환자를 조기발견하여 격리치료하는 것이다. 메르스는 증상이 비특이적이므로 임상적 판단으로 조기진단하기는 어렵다[3]. 따라서 감시체계를 통하여 발열 호흡기 환자의 중동 여행 경력 등을 확인하는 것이 조기진단에 매우 중요한 요인이다.

메르스 의심환자 발생 시 초기 단계에서 빠른 환자 격리, 의료진 보호장구 착용, 검체 의

뢰 등이 신속하게 이루어져야 한다. 첫 확진자가 발생하면 질병관리본부는 감염병 위기 단계를 관심에서 주의 단계로 격상시켜야 한다. 주의단계가 되면 질병관리본부에 본부장 산하 대책본부를 설치하고 메르스 대응을 총괄 지휘하게 된다. 우리나라에서도 2015년 5월 20일 첫 환자를 확진하면서 위기단계를 주의로 격상하고 질병관리본부에 대책본부를 설치하였다[4].

초기 역학조사 원칙

첫 환자에 대한 역학조사는 질병관리본부의 역학조사과에서 시행하게 되는데 이때 매우 신중하고 철저한 역학조사와 이에 따른 적절한 방역 대책을 수립하여야 한다. 특히 그 역학적 특성에 대해 우리가 잘 알지 못하는 신종 감염병인 경우에는 최대한 광범위한 방역대책을 시행하는 것이 안전하다.

역학조사의 첫 단계는 유행 여부를 판단하고 그 크기를 측정하는 것으로 다음 방법을 따른다. 첫째, 환자 또는 의심되는 사례들의 발생 규모를 정확하게 파악한다. 일단 확진되지 않은 환자라도 의심되는 환자들은 모두 몇 명인지를 파악한다. 둘째, 비슷한 질환군이 발생되더라도 이들이 동일질환인지를 확인한다. 통상 역학조사 초기에는 어떤 질병인지 알기 어렵기 때문에 초기 환자들의 면담을 통해서 해당 유행의 사례정의를 내린다. 처음에 확진적 사례정의를 사용하기는 어렵기 때문에 넓은 범위의 사례정의를 정한 후 정보가 더 모이면 차츰 엄격한 사례정의를 사용할 수 있다. 또한 검사실 확진이 나오면 확진 환자, 검사실 확진은 없으나 역학적 연관성과 해당질환의 증상을 보이면 의심 환자로 환자를 분류하여 환자의 규모를 평가한다. 의심 환자 중에서 검사실 확진이 나오면 확진 환자로 분류가 바뀌게 된다

[2,5]. 셋째, 환자 발생 규모가 확인이 되었으면 유행 여부를 판단한다. 유행 여부를 판단할 때는 과거 자료를 기초로 기대되는 발생 수가 얼마인지를 알아야 한다. 만약 장기간의 변동이라면 더욱 과거자료와의 비교가 필요하다. 그러나 신종 감염병과 같이 이전에 우리나라에 환자가 없었거나, 페스트와 같이 과거 장기간 발생한 적이 없지만, 유행의 가능성이 있는 질병은 1명의 환자라도 발생하면 유행의 가능성이 매우 높을 것으로 판단해야 한다.

역학조사의 첫 번째 단계에서 유행여부를 판단하고, 유행의 크기를 추정하여 얼마나 신속한 조치가 필요한지를 결정하게 된다. 또한 유행 원인을 밝히기 위하여 가능한 모든 가검물을 채취하여 분석하고 보관하는 일도 중요하다.

유행 확인 후 조치

역학조사의 첫 번째 단계를 시행하여 유행으로 판단되면 감염병의 확산을 방지하기 위한 방역조치를 수행하여야 한다. 이때 감염병의 종류와 유행의 시기에 따라 방역조치의 우선순위와 내용이 달라지게 된다. 만약 식중독처럼 이미 유행의 원인이 되는 노출이 끝났고, 더 이상의 노출이 없다면 유행 원인으로 의심되는 식품의 보존이나 현장의 보존을 통해서 감염 경로를 밝히기 위한 준비를 하는 것이 좋다. 하지만 사람간 전파가 되는 감염병이고, 유행의 초기라면 신속하고 적극적인 방역조치가 도입되어야 한다. 사람간 전파가 되는 감염병은 환자가 곧 감염원이 되므로 의심환자를 초기에 찾아서 격리 치료하는 것이 중요하다. 또한 질병에 따라서 노출 후 증상이 발생하기까지의 잠복기 동안에 감염전파를 시키는 질병인지, 잠복기 동안에는 감염전파를 시키지

않고 증상 발생 이후에 전염을 시키는 질병인지 등 감염력이 있는 시기와 질병의 자연사를 함께 고려하여 방역조치를 달리하게 된다. 감기나 독감, 홍역 같은 감염병은 증상 발생 전에 이미 전파를 시키기 때문에 노출자를 파악하여 격리조치를 시행하는 것이 매우 어렵고 비효율적이다. 그러나 메르스는 중증급성호흡기증후군(사스)와 같이 잠복기 동안에는 전염을 시키지 않고 증상 발생 이후에 감염력이 있는 것으로 알려져 있어 접촉자를 적극적으로 파악하여 감시조치를 시행하거나 밀접접촉자를 격리조치 하는 것은 질병 확산에 효율적인 방법이 될 수 있다[6,7]. 그러나 이러한 방법을 시행하기 위해서는 확진환자의 동선별 접촉자를 확인하여 방역 관리 대상자를 선정하는 것이 필요하다. 또한 확진자의 동선에서 환경오염이 있을 수 있는 부분에 적절한 소독을 시행하여 추가 감염 가능성을 제거하는 것도 중요하다[6].

이 글은 역학조사에 대한 것이므로 자세한 방역조치에 대한 논의는 포함하지 않을 것이다. 다만 방역조치를 하기 위해서 역학조사에서 확인해야 할 것들은 다음과 같다. 1) 환자의 감염전파 가능기간 확인(메르스는 증상 발생일 이후), 2) 감염전파 가능기간 동안 환자의 동선 파악, 3) 환자의 동선별 접촉자 확인, 4) 환자의 동선에 의료기관이 포함되어 있다면 해당 의료기관에 대한 방역조치가 신속히 이루어지도록 하여 감염확산 가능성 차단, 5) 환자의 주요 동선을 확인한 후에 가능하다면 환자와 직접 인터뷰를 하여 동선을 확인하고, 동선별 접촉자 리스트를 보완 작성. 이때 필요하면 신용카드내역 조회 동의를 받도록 함, 6) 신용카드 조회를 통하여 환자의 동선 기록에서 빠진 부분이 없는지 추가 확인, 7) 환자의 접촉자들을 대상으로 증상발생 여부를 확인하고 만약 증상 발생자가 있다면 첫 환자와 같은 조사를 다시 시작, 8) 필요한 경우 환자의 동선에 따른 CCTV 확인을 통하여 접촉자 누락이 없는지 확인, 9) 필요한 경우 환경 검체 채취 및 검사 수

행, 9) 필요한 경우 의료기관의 시설 점검을 통해서 유행 확산 예방 조치를 수행해야 한다.

중동호흡기증후군 역학조사의 실제

이번 메르스 유행 시 실제 시행되었던 역학조사의 내용을 시간의 흐름과 중요한 변화의 계기가 되었던 사건들을 중심으로 기술해 보았다. 유행 초기와 유행이 확산되면서 기존의 지침과 지식에 부합하지 않은 환자들이 발생하면서 역학조사의 방법이 진화하였고, 방역대상자 선정기준 및 관리방침 등이 변화하였다.

1. 2015년 5월 20일부터 25일까지: 2014 중동호흡기증후군 관리지침에 따라

5월 20일 첫 메르스 환자가 확진되고, 질병관리본부는 밀접접촉자 정의 및 범위 설정, 역학조사를 시행하여야 하는 병의원 설정에 대해 2014년에 발간된 중동호흡기증후군 관리기준[2]을 바탕으로 계획을 수립하였다. 밀접접촉자는 확진 환자 또는 의사 환자와 신체적 접촉을 한 자 또는 환자가 증상이 있는 동안 2 m 이내의 공간에서 1시간 이상 함께 머문 자로 하였다. 이에 따라 의료진 16명에 대해 자택격리 및 능동감시 조치가 취해졌다. 또한 의사환자의 정의에 대해서는 지침 상 임상적, 방사선학적, 조직·병리학적으로 폐실질 질환(예를 들어 폐렴 또는 급성호흡곤란 증후군)이 있는 급성 호흡기 감염자로 하였으나, 실질적으로는 38℃ 이상의 발열을 기준으로 하였다. 병원에 대한 현장역학조사 당시 노출수준을 객관적으로 확인하기 위해서 CCTV 영상을 분석하였다. 범죄수사 등에는 흔히 쓰이는 방법이지만, 우리나라의 감염병 역학조사에서 이런 방법을

적용한 것은 이번이 처음이었다.

2. 5월 25일부터 6월 3일까지: 중동호흡기증후군 대응지침 3판에 따라

5월 23일에 3번째 확진자의 딸인 4번째 환자가 자가 체온측정 결과 37.9℃로 확인하고, 메르스 확진 검사 및 시설격리를 요청하였으나 관계 당국이 이를 거부하였다. 이후 5월 26일에 확진을 받게 되었다. 또한 같은 날에 발단 환자를 5분 이내로 진료하였던 의사가 5번째로 확진을 받았다. 이에, 의사 환자의 기준을 37.5℃로 낮추고, 발열이 아닌 근육통, 오한, 설사 등의 증상도 폭넓게 고려해야 한다는 의견이 새 지침에 반영되었다. 그리고, 밀접 접촉에 대한 시간의 기준에 대해서도 1시간 이상을 적용하는 것이 더 이상 합리적이지 않다고 판단하여 새 지침에는 시간 기준이 없다. 지침이 새로 만들어지고 현장에 배포되기까지는 시간적인 간격이 있기 때문에 역학조사관들을 이러한 사항들을 즉각적으로 소통하여 현장 업무에 반영하였다.

3. 6월 3일부터 6월 7일까지: 중동호흡기증후군 대응지침 3-2판에 따라

5월 28일에는 첫 환자와 같은 병원에 입원하였으나 다른 병실에 있어서 밀접접촉자로 분류하지 않았던 환자 중에서 메르스 확진자(6번째 확진자)가 나왔다. 보건 당국에서는 첫 환자와의 밀접 접촉이 있었는지 확인하기 위하여 평택성모병원에 대한 전반적인 재조사에 착수하였다. 또한 5월 30일에는 민관 합동 "메르스 감염경로 역학조사위원회"가 발족되었다. 역학조사 내용에는 병원 CCTV 재분석, 병원 도면에 추후 발생하는 환자들의 공간적 분포를 확인하는 작업, 전체

의료진 및 환자들의 명단 확보와 노출에 대한 재평가 및 밀접접촉자 재분류, 병동 내 환기시스템에 대한 환경조사, 환경검체수거 및 검사 등이 포함되었다. 전반적인 재조사에도 밀접접촉을 확인하기 어려웠고, 비슷한 환자들이 추가적으로 발생함에 따라 기존의 비말에 의한 밀접 접촉의 전파 경로만을 생각하기에는 무리가 있다고 판단하여 질병의 전파 경로 규명을 위한 병동환경에 대한 기류 확산 검사, 의료진에 대한 심층조사 등을 추가적으로 시행하였다. 지침에도 이와 같은 부분이 반영 되어 발열과 호흡기 증상이 있으면서 증상 발생 14일 이내에 메르스가 유행한 의료기관에 직원, 환자, 방문자로 있었던 자를 의사환자의 정의 부분에 추가하였다. 또한, 6월 초부터는 건강보험심사평가원 인력들이 역학조사 지원에 참여하게 되었다. 이는 6월 5일에 평택성모병원을 공개하면서 환자들의 병원 방문력을 일선 의료기관에서 확인할 수 있도록 지원하기 위한 것도 있었고, 역학조사에서 필요한 환자들의 병의원 방문력을 확인하기 위한 목적도 있었다. 이는 역학조사가 진행되면서 환자들의 진술에만 의존하다 보니 실제 방문하였던 의료기관들을 놓치게 되는 사례가 발생하면서 건강보험심사평가원의 의약품안심서비스 자료를 조회하여 환자들의 정확한 병의원 및 약국 방문력을 확인하여 역학조사에 활용하고자 한 것이다. 이 역시 기존의 역학조사에서는 사용하지 않았던 새로운 방법이었다.

4. 6월 7일부터 현재까지: 중동호흡기증후군 대응지침 3-3판에 따라

6월 7일에는 메르스 환자 발생 및 경유 병원을 전면공개하고, 해당 병의원에 메르스 환자가 있었던 기간 동안 방문하였던 사람들에게 증상 발생 시 콜센터로 연락하도록 조치하였다.

또한 대응지침 3-3판을 발간하였다[8]. 중동호흡기증후군 대응지침 3-2판 이후로 밀접접촉자나 환자 정의에 대한 변함은 없었다. 다만, 이후 개정된 지침은 사태가 장기화됨에 따라 확진 환자들의 치료나 완치 판정, 자가격리자들의 격리 해제, 환자들의 사망 시 처리문제 등 파생적인 문제들에 대한 지침들이 대거 포함되었다. 이 즈음부터는 평택성모병원의 유행 건을 교훈으로 삼아 대청병원이나 건양대학병원에 대한 코호트격리도 적극 시행할 수 있었다. 또한 환자의 역학조사 당시의 진술과 주변인들의 진술이 일치하지 않아 명확한 동선파악에 어려움이 있었던 환자들이 늘어나면서 이를 보완하기 위해 핸드폰 기지국 위치추적을 통한 진술의 근거 확보와 신용카드 사용내역을 확인하는 등의 조치가 추가 병행되었다. 또한 외부로 여행을 갔던 환자의 동선을 추적하기 위하여 환자가 여행하면서 빌렸던 렌터카의 차량번호를 통한 동선 파악도 시행하였다. 이러한 방법들 역시 기존의 역학조사에서는 사용하지 않았던 방법이다.

역학조사에서 부딪히는 문제

1. 역학조사관의 역할

역학조사관은 역학조사를 수행하면서 확진자의 접촉자를 파악하여 접촉수준에 따른 방역대상자의 기준을 정한다. 접촉 수준별 대상자를 찾고 이들에게 개별 연락하여 증상여부를 확인하고, 필요하면 검사를 시행하고, 격리조치, 능동감시 등의 추적관리 등의 방역조치는 해당지역의 시도보건과와 보건소에서 수행한다. 하지만 이러한 역할이 때로 명확하게 구분하기 어려운 경우

가 많다. 이번 메르스 유행처럼 병원에서 유행이 발생한 경우 접촉수준에 따른 방역대상자의 기준을 역학조사관이 결정한다고 해도 병의원에서 협조해주지 않으면 보건소에서 대상자를 격리하거나 추적관리하기가 매우 어렵다.

또한 시급한 방역조치를 위하여 환자 발생 병원의 일시적 업무정지 등이 필요할 때 이를 명령할 권한이 역학조사관에게 법적으로 부여 되어있지 않고, 대규모 병원의 경우 업무정지에 따른 일반 환자들의 치료 권리가 침해되는 문제가 매우 커서 역학조사관 단독으로 결정하기는 매우 어렵다. 이번 메르스 유행에서도 이러한 문제가 계속 제기되었다. 그 외에도 역학조사 시에 도출되는 여러 문제를 실시간으로 논의하여 의사결정하는 시스템이 없어서 의사결정이 늦어지거나, 논의되지 못하고 역학조사관 혼자 결정해야 하는 문제들이 발생하였다.

2. 개인정보 보호

역학조사에서 밀접접촉자를 선별하여 방역조치를 취하기 위해서는 환자와 접촉자들의 의무기록뿐 아니라 사적인 생활 영역까지도 조사해야 하는 경우가 있다. 이때에 개인정보보호제도와 모순이 없이 역학조사를 수행할 수 있는 규정이 없어서 역학조사에 많은 어려움이 나타났다. 또한 이번 메르스 유행에서는 증상이 나타난 이후 확진이 될 때까지 짧게는 하루 이틀에서 길게는 일주일 이상까지 일상생활을 한 환자들이 많이 있었다. 그런 경우 그 기간 동안 접촉자들을 찾기 위해서는 환자의 인터뷰에만 의존해서는 많은 부분을 놓치게 된다. 환자들이 모두 다 기억할 수도 없으며, 환자의 상태가 면접이 어려울 정도로 위중한 경우도 있다. 때로는 환자들이 개인

적인 이유로 접촉력을 숨기기도 한다. 이번 메르스 유행에서도 이러한 원인으로 접촉자를 놓치게 되고, 그렇게 방역망에서 빠진 접촉자가 다시 확진이 되어 커다란 유행 위험을 가져오기도 하였다. 따라서 건강보험심사평가원 자료를 활용하여 환자들의 의료기관 이용 내역을 조회하고, CCTV, 휴대폰, 신용카드 사용내역 조회 등을 동원하여 환자들의 동선을 파악하였다. 이때에 CCTV 확인을 위해서는 전문가의 도움이 필요하고, 휴대폰 위치추적과 신용카드 이용내역 조회에는 환자본인 및 보호자, 동거인 등 까지도 필요한 경우가 있다. 그런데 이는 모두 당사자의 동의가 있어야만 한다. 이러한 과정에서 개인의 인권, 정보보호 등을 최대한 유지하면서 신속하게 역학조사의 목적을 달성할 수 있는 방안이 마련되어야 할 것이다.

3. 역학조사관 포함 조사인력의 감염위험 문제

이번 메르스 유행처럼 병원에서 환자가 발생한 경우 해당 병의원이 이미 오염된 상태이고, 환자와의 면접조사도 밀접접촉에 해당되므로 역학조사관은 감염을 철저히 예방할 수 있는 준비가 된 후에 조사를 시행하여야 한다. 그런데 이번 유행 역학조사 초기에는 역학조사관에게 레벨D 방호복도 지급되지 않았고 심지어 해당 병원 내에 역학조사팀이 설치되기도 하였다. 만약에 역학조사관을 포함한 역학조사인력의 감염이 발생한다면 역학조사팀 전체가 격리조치 되어야 하는 초유의 사태가 발생하게 되며 이는 곧 역학조사의 공백으로 이어지게 되므로 이들의 감염예방은 무엇보다 중요하다고 할 수 있다.

4. 방역조치에 따른 보상체계 미비

역학조사를 통하여 환자와 밀접접촉한 것으로 확인되면 개인의 경우에 자가격리나 능동 감시 등의 방역조치를 받게 되고, 병의원을 포함한 시설에서는 방역지침에 따라 일시폐쇄 등의 조치가 따르게 된다. 그런데 이러한 조치에 따른 개인과 기관의 피해에 대하여 어떠한 보상이 주어지는지에 대한 지침이 없었다. 이는 곧 개인과 기관의 비협조로 이어져 방역조치의 실효성이 떨어지는 결과를 낳았다.

5. 중앙과 지역 시도보건과, 보건소와의 유기적 협조체계

이번 메르스 유행은 빠른 시간에 질병이 여러 지역의 병원들로 확산되면서 관련 지자체와 중앙의 유기적 협조가 무엇보다 중요하였다. 그런데 중앙에서 초기에 정보공개를 늦추고 검사를 지자체 보건환경연구원으로 신속하게 이양하지 못하면서 지자체의 대응도 늦어지고 불협화음을 초래하게 되었다.

역학조사 강화방안

앞에서 열거한 여러 문제점들이 해결되어야만 역학조사가 제대로 시행될 수 있고 그 역할을 다 할 수 있을 것이다. 향후 우리나라에서 역학조사를 지금보다 한층 발전시키기 위한 몇 가지 제언을 하고자 한다.

1. 관련 법령 검토 및 수정 필요

역학조사에 대하여 감염병법 제2조 제17호에서는 감염병환자, 감염병의사환자 또는 병원체보유자(이하 '감염병환자 등')가 발생한 경우 감염병의 차단과 확산 방지 등을 위하여 감염병환자 등의 발생 규모를 파악하고 감염원을 추적하는 등의 활동과 감염병 예방접종 후 이상반응 사례가 발생한 경우 그 원인을 규명하기 위하여 하는 활동으로 정의하고 있다.

시행령 제12조 제1항에서는 역학조사의 내용을 1) 감염병환자등의 인적 사항, 2) 감염병환자 등의 발병일 및 발병 장소, 3) 감염병의 감염원인 및 감염경로, 4) 감염병환자 등에 관한 진료기록, 5) 그 밖에 감염병의 원인 규명과 관련된 사항으로 명시하고 있다.

또한 역학조사 방법에 대하여 시행령 제14조, 별표 1에 1) 설문조사·면접조사, 2) 인체검체 채취 및 시험, 3) 환경검체 채취 및 시험, 4) 감염병 매개 곤충 및 동물의 검체 채취 및 시험, 5) 의료기록 조사 및 의사 면접 등으로 기술하고 있다.

그런데 이러한 법령에는 이번 메르스 유행처럼 역학조사가 곧 방역조치로 이어지는 매우 급박한 상황에 필요한 내용은 포함되어 있지 않다. 예를 들어 신속한 방역조치를 위하여 감염병 환자의 접촉자를 파악하기 위하여 개인정보보호법에도 불구하고 환자의 동선파악에 필요한 다양한 개인정보 확인 조치(CCTV 확보 및 분석, 휴대폰 위치추적 및 신용카드 조회 등)를 허가하는 문구는 없다. 또한 향후에도 질병의 종류와 특성에 따라 역학조사의 내용과 범위, 방법 등이 달라질 수 있는데 이에 대하여 유연하게 대응할 수 있도록 명시할 필요가 있다.

2. 역학조사 전문인력 보강 및 역할강화

이번 메르스 유행을 통하여 현재의 역학조사관의 지위나 제도의 미비점에 대해서 많은 비판이 있었다. 이에 국회에서는 감염병의 예방 및 관리에 관한 법률을 개정하면서 역학조사관 제도도 개정하였다. 개정법률은 7월 6일자로 공포되면서 바로 시행되었고, 이중에서 역학조사관 제도는 2016년 1월부터 시행될 예정이다. 역학조사관 인력 보강에 대해서는 "제60조의 2(역학조사관) ① 감염병 역학조사에 관한 사무를 처리하기 위하여 보건복지부 소속 공무원으로 30명 이상, 시·도 소속 공무원으로 각각 2명 이상의 역학조사관을 둔다. 다만, 시·도지사는 역학조사에 관한 사무를 처리하기 위하여 필요한 경우 시·군·구에도 역학조사관을 둘 수 있다"고 명시하였고, 역학조사관의 권한으로는 "③ 역학조사관은 감염병의 확산이 예견되는 긴급한 상황으로서 즉시 조치를 취하지 아니하면 감염병이 확산되어 공중위생에 심각한 위해를 가할 것으로 우려되는 경우 일시적으로 제47조 제1호 각 목의 조치를 할 수 있다"고 명시하였다. 이때 제47조 제1호 각 목의 조치는 "감염병환자 등이 있는 장소나 감염병병원체에 오염되었다고 인정되는 장소에 대한 가) 일시적 폐쇄, 나) 일반 공중의 출입금지, 다) 해당 장소 내 이동제한, 라) 그 밖에 통행차단을 위하여 필요한 조치"를 말한다[9].

이러한 법령의 개정이 늦게나마 이루어진 것은 다행이다. 그러나 이 법령이 목적한 바 효력을 발휘하기 위해서는 역학조사관을 교육하는 기구가 신설되어 역량 있는 역학조사관을 지속적으로 교육시킬 필요가 있으며, 교육 후에도 지속적으로 역학조사관의 경험을 활용할 수 있는

시스템이 마련되어야 한다. 그리고 능력 있는 전문가들이 역학조사관에 많이 지원하도록 하려면 역학조사관에 대한 획기적인 처우개선이 있어야만 한다. 이러한 역학조사관 강화방안에 대해서는 앞으로도 여러 분야 전문가들이 모여 다양한 논의를 통해서 제도의 구체적 틀을 마련해야 할 것이다.

3. 신종 감염병 연구센터 필요

이번 메르스 유행을 겪으면서 우리나라가 신종 감염병 대응에 준비가 되어있지 않음을 전 세계가 알게 되었다. 또한 IT강국으로 알려진 한국에서 디지털 시대를 살고 있음에도 불구하고 역학조사는 아직도 공문서를 보내서 협조를 구하고 역학조사서를 종이에 기록하고 워드로 옮겨서 정리하여 보고할 때까지 기다리고 있는 구시대적 틀을 그대로 유지하고 있었다. 이러한 시스템은 유행이 거의 끝나가는 시점에 역학조사가 시작되는 경우에는 큰 문제가 없었으나 이번 유행처럼 역학조사가 곧 방역조치를 결정짓고, 유행의 확산을 신속히 제어하는 역할을 하는 급박한 경우에는 매우 효율이 떨어지는 것이었다. 또한 역학조사 결과를 전문가뿐 아니라 대중에게 바로 알려야 하는데 위험소통이 제대로 되지 않아 과도한 불안과 공포가 확산되었으며 그 결과 불필요한 학교 폐쇄가 전국에서 시행되기도 하였다. 이러한 문제를 해결하기 위하여 신종 감염병 연구센터를 설립하여 다양한 분야의 전문가가 이러한 감염병 유행에 대비할 수 있는 상시 시스템을 마련하는 것이 필요하다. 이러한 기구에서 전세계 질병 동향을 면밀히 관찰하고 국내 대응을 준비하도록 하여야 한다. 또한 전자의무기록를 포함하여 다양한 개인 정보원(CCTV, 내비게이션, 신용카

드, 과거 병원이력 등)을 활용하여 역학조사를 시행하는 디지털 역학조사의 체계도 마련하고, 역학조사 내용을 전문가와 정책결정가가 실시간 공유할 수 있는 시스템도 마련하여야 할 것이다. 또한 위험소통전문가가 모든 역학조사의 단계별로 전문가, 일반인, 교육계 등과 소통할 수 있도록 준비하는 것도 필요하다.

결론

우리나라는 이번 메르스 유행을 통하여 신종 감염병에 대한 준비 부족과 이로 인한 다양한 문제점들을 고스란히 드러내었다. 이러한 문제점들을 근본적으로 고치지 못한다면 제2, 제3의 메르스 유행은 언제라도 다시 일어날 수 있을 것이다. 빌 게이츠는 21세기 인류를 위협하는 가장 큰 적은 핵이 아니라 신종 전염병이라고 일찍이 경고하였다[10]. 질병에 대한 최선의 치료는 예방인 것처럼 우리나라도 이번 위기를 기회로 삼아 신종 감염병 유행에 의한 사회경제적 피해를 선제적으로 예방할 수 있도록 대비를 굳건히 하여야 할 것이다.

REFERENCES

1. Korean Society of Preventive Medicine. Preventive medicine and public health. 2nd ed. Seoul: Gyechuk Munhwasa; 2015.

2. Korea Centers for Disease Control and Prevention. Guideline for management of MERS. Cheongju: Korea Centers for Disease Control and Prevention; 2014.

3. World Health Organization. Clinical management of severe acute respiratory infection when Middle East respiratory syndrome coronavirus (MERS-CoV) infection is suspected [Internet]. Geneva: World Health Organization; 2015 [cited 2015 Jul 20]. Available from: http://www.who.int/emergencies/mers-cov/en/.

4. Korea Centers for Disease Control and Prevention. Middle East respiratory syndrome information 2015 [Internet]. Cheongju: Korea Centers for Disease Control and Prevention; 2015 [cited 2015 Jul 27]. Available from: http://mers.go.kr/mers/html/jsp/main.jsp.

5. Madani TA. Case definition and management of patients with MERS coronavirus in Saudi Arabia. Lancet Infect Dis 2014;14:911-913.

6. Centers for Disease Control and Prevention. Infection prevention and control recommendations for hospitalized patients with Middle East respiratory syndrome coronavirus (MERS-CoV) [Internet]. Atlanta: Centers for Disease Control and Prevention; 2015 [cited 2015 Jul 27]. Available from: http://www.cdc.gov/coronavirus/mers/infection-prevention-control.html.

7. World Health Organization. Infection prevention and control of epidemic and pandemic prone

acute respiratory infections in health care: WHO guidelines [Internet]. Geneva: World Health Organization; 2015 [cited 2015 Jul 5]. Available from: http://www.who.int/csr/bioriskreduction/infection_control/publication/en/.

8. Korea Centers for Disease Control and Prevention. Guideline for management of MERS [Internet]. Cheongju: Korea Centers for Disease Control and Prevention; 2015 [cited 2015 Jul 20]. Available from: http://www.cdc.go.kr/CDC/info/CdcKrHealth0289.jsp?menuIds=HOME001-MNU1132-MNU1013-MNU1913&cid=63682.

9. Law on Infectious Diseases Prevention and Control, No. 13392 (July 6, 2015).

10. McSpadden K. Bill Gates thinks this is the deadliest threat to humankind. Time. 2015 May 28 [cited 2015 Jul 31]. Available from: http://time.com/3899414/bill-gates-disease-epidemic-ebola-threat-to-humanity-disaster/#3899414/bill-gates-disease-epidemic-ebola-threat-to-humanity-disaster/.
